# Pharmacovigilance in healthcare education: students’ knowledge, attitude and perception: a cross-sectional study in Saudi Arabia

**DOI:** 10.1186/s12909-020-02116-2

**Published:** 2020-07-02

**Authors:** Monira Alwhaibi, Ghaida Alhindi, Majd Alshamrani, Maryam Bin Essa, Noha A. Al Aloola, Tariq M. Alhawassi

**Affiliations:** 1 Department of Clinical Pharmacy, College of Pharmacy, King Saud University, Riyadh, 11149 Saudi Arabia; 2 Medication Safety Research Chair, College of Pharmacy, King Saud University, Riyadh, Saudi Arabia

**Keywords:** Adverse drug reactions, Attitudes, Healthcare students, Knowledge, Perception, Pharmacovigilance

## Abstract

**Background:**

Medication safety and pharmacovigilance (PV) remains as an important discipline worldwide. However, there is a significant lack of knowledge of PV and adverse drug reaction (ADR) reporting among students in the healthcare field. Thus, this study is aimed to measure knowledge, attitude, and perceptions and compares it between healthcare students (i.e., medicine, dentistry, and nursing).

**Methods:**

A cross-sectional study involving 710 undergraduate healthcare students from different universities in Saudi Arabia was conducted. A validated structured pilot-tested questionnaire was administered to the participants to assess their knowledge, attitude, and perceptions towards PV and ADRs reporting. Descriptive statistics were used to describe the study findings. Data were analyzed using SPSS version 21.

**Results:**

Overall, the study found that 60.8 and 40.0% of healthcare students correctly defined PV and ADRs respectively. Most students showed positive attitudes and perceptions towards PV and ADRs reporting. PV knowledge, attitude, and perceptions towards PV were significantly higher among pharmacy students as compared to other healthcare students. Only 39% of healthcare students revealed that they have received any form of PV education and 49% of them indicated that PV is well covered in their school curriculum. Pharmacy students are more trained in their schools to report and have performed ADRs reporting in their school as compared to other healthcare students.

**Conclusions:**

Pharmacy students have better knowledge, attitude, and perception towards PV and ADR reporting in comparison to other healthcare students. The study clearly describes the need for integrating pharmacovigilance education in Saudi healthcare schools’ curriculums to prepare them for real-world practices and workplaces.

## Background

Medication safety and pharmacovigilance remains an important subject and discipline worldwide. The World Health Organization (WHO) defines pharmacovigilance (PV) as “the science and activities relating to the detection, assessment, understanding and prevention of adverse effects or any other drug-related problem” [1]. Worldwide, around 137 countries were part of the WHO PV Programme [2]. The Saudi Food and Drug Authority (SFDA) established a pharmacovigilance system that functions under the National Pharmacovigilance and Drug Safety Center to monitor drug safety in the Saudi market. Nowadays, the NPC conducts numerous pharmacovigilance activities, for instance, Adverse Drug Events (ADE) evaluation, vaccine safety evaluation, signal detection, assessment of periodic safety update reports, risk assessment and analysis.

One of the major challenges that pharmacovigilance programs face worldwide is underreporting [3]. Saudi Arabia together with other ten Middle Eastern countries only contributed 0.6% to the global safety database reporting (i.e., the WHO’s Vigibase) [4]. This fact indicates that ADRs (adverse drug reactions) underreporting is a major concern in Saudi Arabia. Underreporting of ADRs could be attributed to the knowledge deficit [4], lack of training or education that health care providers have regarding PV and the safety of medications [5, 6].

The lack of knowledge of PV and ADRs reporting among HCPs is most likely due to the unavailability of PV as a subject of study in healthcare schools (i.e. medicine, pharmacy, dentistry, nursing, etc.) [7–10]. In fact, a systematic review of 27 studies investigated the PV and ADR-reporting competencies of healthcare students. This review found a lack of knowledge on how to report. Also, students felt inadequately qualified to report ADRs or to perform PV [11]. Further, there is some evidence that supports a variation in knowledge of PV among healthcare students; pharmacy students had a significantly higher knowledge of PV compared to students from other healthcare schools [12, 13].

The above-mentioned evidence indicates that there is a significant lack of knowledge of pharmacovigilance among students in the healthcare field and this knowledge may vary by the healthcare field. Also, there are many published studies in this area, most of the published studies did not compare healthcare students that need to be educated about pharmacovigilance and ADR reporting as the will encounter in their practice and all need to report the ADRs. The findings of this study can help in identifying educational gap among healthcare students which is essential for the design of educational program which can untimely help in promoting the PV environment and safe practices among future healthcare professionals. Therefore, the objective of this study was to estimate and compared the knowledge, attitude, and perceptions of pharmacovigilance among students from different healthcare colleges in Saudi Arabia.

## Methods

### Study design and sample

This is a questionnaire based cross-sectional study that was conducted by administrating the questionnaire to actively enrolled healthcare students (i.e. medicine, pharmacy, nursing, and dentistry) in Saudi Arabia which has around 23 government colleges and seven private colleges [14]. The study protocol was approved by the Research Center of Medical College of King Saud University and its Ethical Committee (No. E-19-4004).

### Questionnaire development

The questionnaire was constructed after an extensive review of the literature to identify existing instruments [15–17]. An initial draft of the questionnaire was made and was comprised of 1) Demographics, 2) PV and ADRs knowledge, attitude, reporting perception, and 3) PV and ADRs actual practice in healthcare colleges (Additional file 1).

### Validation of the questionnaire

A group of investigators (*n* = 5) reviewed the survey and the instruments for the relevance, content, clarity, and ease of understanding of the questions. The observations and comments of the researchers were taken into account. Items included in the questionnaire cover all the aspects of knowledge, attitude and perception being measured, and each item was linked to the objectives of this study as well. Thus, it indicates content and face validity.

The survey was pilot-tested among a purposive sample of healthcare students of diverse healthcare schools (*n* = 22) to evaluate the appropriateness of the questions to the target sample. The responses from these participants were not included in the final analysis. The internal consistency “reliability” of the items was estimated using Cronbach’s alpha coefficient. The internal consistency of the questionnaire items was good (Cronbach’s alpha was 0.6 for knowledge, 0.9 for attitude, and 0.7 for perception). Item analysis was conducted for the knowledge items using item Difficulty and Discrimination. The difficulty of an item is the proportion of the persons who answer a test item correctly, with a higher score indicating a lower difficulty. The items demonstrated low to medium difficulty (0.02–0.86). Also, the items demonstrated a higher discrimination index (0.1–0.5) which indicates good quality of the items.

### Data collection

The survey then was distributed online via Google forms and the link was sent via email and social media explaining the purpose of the study. Each eligible participant was asked to complete an informed consent form that explained the purpose of the study and their right to withdraw from the study at any time before proceeding to complete an anonymous online survey. The survey response rate was 65%. Non-responders include those who did not reply to the online survey, those who clicked on the link but did not respond and those who did not complete the survey.

### Measures

#### Dependent variables

The dependent variables of this study were the PV and ADRs knowledge, attitude, reporting perception, and actual practice in healthcare colleges. Knowledge about pharmacovigilance and ADR reporting was measured by asking students to select the correct answer for 11 questions. A score of 1 was given for each correct answer and 0 for the wrong answer. The maximum score obtainable was 11 and the minimum was 0. Perceptions of students toward pharmacovigilance activities and ADR reporting were measured by asking students to select an answer to three questions. The maximum score obtainable was three and the minimum was 0. The attitude of students toward pharmacovigilance and ADR reporting was measured using a 5-point Likert-scale (1 = strongly agree to 5 = strongly disagree). The maximum score obtainable was 20 and the minimum was 4. Lower scores indicate a positive attitude towards PV and ADR reporting.

#### Independent variables

Independent variables comprised of participants’ demographics such as age, gender, year of study (4th, 5th, 6th, and intern), and type of college (private, governmental).

### Statistical analyses

Descriptive statistics were used to describe the study findings. Mean ± standard deviation (SD) and frequency (%) were used to describe continuous and categorical variables. Chi-square tests were used to conduct bivariate analyses on categorical and continuous variables. A *p*-value of less than 0.05 was considered statistically significant. All statistical analyses were carried out using SPSS software version 21.0.

## Results

### Demographic information

The total number of healthcare students who participated was 710; around 40% (*n* = 282) were pharmacy students, 29% (*n* = 208) medical students, 17% (*n* = 123) dentistry students, and 14% (*n* = 97) nurse students. Around 41% were in the 4th year, 18% in the 5th year, and 14% in the 6th year while 27% were in the internship year. The majority were female students (65%). The summary of the demographic information is presented in Table 1.

**Table 1:** Description of the study sample

	Total	Medicine	Pharmacy	Dentistry	Nursing
	N (%)	N(%)	N(%)	N(%)	N(%)
**Total**	710 (100)	208 (29.3)	282 (39.7)	123 (17.3)	97 (13.7)
**Gender**					
Male	246 (38.6)	98 (13.8)	74 (10.4)	41 (5.8)	33 (4.6)
Female	464 (65.4)	110 (15.5)	208 (29.3)	82 (11.5)	64 (9.0)
**Year of Study**					
4th year	291 (41.0)	79 (11.1)	108 (15.2)	49 (6.9)	55 (7.7)
5th year	125 (17.6)	38 (5.4)	59 (8.3)	28 (3.9)	0 (0.0)
6th year	102 (14.4)	59 (8.3)	0 (0.0)	32 (4.5)	0 (0.0)
Intern	192 (27.0)	32 (4.5)	115 (16.2)	14 (2.0)	42 (5.9)
**Type of College**					
Private	80 (11.3)	26 (3.7)	23 (3.2)	21 (3.0)	10 (1.4)
Governmental	630 (88.7)	182 (25.6)	259 (36.5)	102 (14.4)	87 (12.3)

Note: Study sample comprised of 710 healthcare students; 282 pharmacy students, 208 medical students, 123 dentistry students, and 97 nurse students

### Knowledge about Pharmacovigilance and ADRs reporting

The study found that 60.8 and 40.0% of healthcare students correctly defined PV and ADRs respectively. Moreover, PV knowledge was higher among pharmacy students as compared to other healthcare students (i.e., medicine, dentistry, and nursing). For example, a significantly higher percentage of pharmacy students correctly defined pharmacovigilance knowledge and ADRs as compared to other healthcare students (*P*-value< 0.0001). Besides, a significantly higher percentage of pharmacy students correctly identified the governmental monitoring agency and vigilance system in Saudi Arabia as compared to other healthcare students (*P*-value< 0.0001). Additional details about the PV knowledge comparison between healthcare students are shown in Table 2.

**Table 2:** Healthcare Students’ knowledge of Pharmacovigilance and ADR reporting

Q. No		% Correct Answers					
		Total	Medicine	Pharmacy	Dentistry	Nursing	***P***-value
**1**	**Definition of PV?**	60.8	16.9	26.9	10.3	6.8	0.008
**2**	**The functions of PV?**	65.2	18.6	30.0	10.4	6.2	< 0.0001
**3**	**Definition of ADR?**	40.0	12.5	13.7	6.9	6.9	0.032
**4**	**ADRs classified based on**	70.6	21.7	29.4	13.1	6.3	< 0.0001
**5**	**The scale used to assess the cause of ADRs named**	35.8	12.1	13.9	5.1	4.6	0.154
**6**	**Hypersensitivity reactions are … … to ADRs**	80.1	23.0	32.8	13.5	10.8	0.598
**7**	**The governmental monitoring agency for ADRs in Saudi Arabia is**	63.4	17.0	28.5	11.1	6.8	< 0.0001
**8**	**Saudi Arabia Vigilance system**	73.0	18.2	33.4	13.2	8.2	< 0.0001
**9**	**Which type of ADRs should be reported?**	78.7	21.4	33.3	14.8	10.3	0.03
**10**	**ADRs that should be reported are related to**	69.9	20.7	30.1	12.4	6.6	< 0.0001
**11**	**Before reporting ADR, conformation that ADR is related to a particular drug is**	88.3	26.8	35.4	14.8	11.4	0.158

Note: Study sample comprised of 710 healthcare students; 282 pharmacy students, 208 medical students, 123 dentistry students, and 97 nurse students

The percentages of responses indicating agreement and strong agreement were combined. P-value derived from the chi-square test

*Abbreviations*: *ADR* Adverse drug reaction, *PV* Pharmacovigilance

### Attitude towards Pharmacovigilance and ADRs reporting

Attitudes of healthcare students were mostly positive; 95.1 and 94.1% agreed that reporting adverse drug reaction makes a significant contribution to the reporting system and patient safety respectively. Around 87.9% of students agreed that reporting adverse drug reactions should be made compulsory for all health care professionals.

Positive attitude towards PV was significantly higher among pharmacy students as compared to other healthcare students (Table 3). For example, 35.2% of pharmacy students agreed that ADRs reporting is their responsibility as compared to 23.4% for medicine, 15.8% for dentistry, and 10.8% for nursing students. Moreover, 39.4% of pharmacy students agreed that ADR reporting should be made compulsory for all healthcare professionals as compared to 25.2% for medicine, 16.1% for dentistry, and 11.0% for nursing students.

**Table 3:** Healthcare Students’ Attitudes Toward Pharmacovigilance and ADR reporting

Q. No		% Agreed					***P***-value
		Total	Medicine	Pharmacy	Dentistry	Nursing	
**1**	**Reporting ADR make a significant contribution to reporting system**	95.1	26.9	38.0	17.2	13.0	< 0.0001
**2**	**Reporting ADR make a significant contribution to patient safety**	94.1	27.7	37.4	17.0	11.8	< 0.0001
**3**	**Reporting ADR is a responsibility of me**	85.2	23.4	35.2	15.8	10.8	< 0.0001
**4**	**Reporting ADR should be made compulsory for all healthcare professionals**	87.9	25.2	39.4	16.1	11.0	< 0.0001

Note: Study sample comprised of 710 healthcare students; 282 pharmacy students, 208 medical students, 123 dentistry students, and 97 nurse students

The percentages of responses indicating agreement and strong agreement were combined. P-value derived from the chi-square test

*Abbreviation*: *ADR* Adverse drug reaction

### Perception of Pharmacovigilance and ADRs reporting

The study found that less than 50% of healthcare students perceive that they are adequately prepared to report ADRs. Around 90% of students believed that PV and ADR reporting system education is needed in their schools, and 85% showed a willingness to participate in such education. Table 4 shows that a significantly higher percentage of pharmacy students have better perceptions regarding the need for PV education and willingness to participate in PV education.

**Table 4:** Healthcare Students’ Perception of PV and ADR Practice

Q. No		Correct Answers (%)					
		Total	Medicine	Pharmacy	Dentistry	Nursing	***P***-value
**1**	**Do you feel you are adequately prepared to report ADR in your future practice?**	42.7	7.9	21.1	5.1	8.6	< 0.0001
**2**	**Do you believe that all medical students’ need education about PV and ADR reporting system?**	89.6	25.4	36.8	15.9	11.5	0.045
**3**	**If you offered an opportunity to undertake education in PV and ADR reporting system, would you be willing to participate?**	85.1	23.7	36.1	15.4	10.0	< 0.0001

Note: Study sample comprised of 710 healthcare students; 282 pharmacy students, 208 medical students, 123 dentistry students, and 97 nurse students

The percentages of responses indicating correct answers to the perception questions. P-value derived from the chi-square test

*Abbreviations*: *ADR* Adverse drug reaction, *PV* Pharmacovigilance

### Current PV and ADRs practice

Only 39% of healthcare students revealed that they have received any form of PV education and 49% indicated that PV is well covered in their school curriculum (Table 5). Besides, only 41% of participants indicated that students in their college trained to report ADR, and 56% indicated that students can perform ADRs reporting during their clerkship. Pharmacy students are more trained in their schools to report and have performed ADRs reporting in their school as compared to other healthcare students.

**Table 5:** Healthcare Students’ Current PV and ADR Practice

Q. No		Correct Answers (%)					
		Total	Medicine	Pharmacy	Dentistry	Nursing	***P***-value
**1**	**Pharmacovigilance is well covered in your college curriculum**	49.0	9.0	23.8	6.8	9.4	< 0.0001
**2**	**Students in your college trained on how to report ADR**	41.0	5.8	22.3	5.2	7.7	< 0.0001
**3**	**Students in your college can perform ADR reporting during their clerkship**	56.1	10.3	28.6	8.2	9.0	< 0.0001
**4**	**Have you received some form of PV education previously?**	39.4	7.6	19.9	5.9	6.1	< 0.0001

Note: Study sample comprised of 710 healthcare students; 282 pharmacy students, 208 medical students, 123 dentistry students, and 97 nurse students

The percentages of responses indicating correct answers to the perception questions. P-value derived from the chi-square test

*Abbreviations*: *ADR* Adverse drug reaction, *PV* Pharmacovigilance

## Discussion

This study was conducted to evaluate and compare knowledge, attitude and perceptions between healthcare students in their fourth, fifth, sixth and internship years in Saudi Arabia, were during these years most students receive training and education about PV and ADRs. In this study, around two-thirds of healthcare students know about PV and ADRs. Attitudes of healthcare students were mostly positive towards the importance of reporting adverse drug reactions to make a significant contribution to the reporting system and that reporting ADRs should be made compulsory for all health care professionals. The majority of healthcare students believed that PV and ADR reporting system education is needed in their schools.

Pharmacy students were found to have better knowledge, attitude and perceptions regarding pharmacovigilance and ADR reporting in comparison to medical, dentistry and nursing students, a noteworthy finding of this study. This finding is somehow expected, pharmacy students in this study reported more training in their schools to report and have performed ADRs reporting and have more exposure to the topic of pharmacovigilance and ADR reporting in their education as compared to other healthcare students. This finding is consistent with two published studies comparing pharmacy and medicine students. Sivadasan et al. conducted a study among pre-final and final year medicine and pharmacy students in Malaysia. The authors found that pharmacy students have better knowledge, awareness and understanding towards PV and ADR reporting compared to medical students [12]. The second study compared PV knowledge among final-year pharmacy and medical students in Pakistan. The study reported that pharmacy students exhibited more knowledge and positive attitudes regarding their ability to handle and report ADRs than medical students [13]. In our study, more medicine and pharmacy students felt adequately prepared to report ADR in their future practice than the reported findings by Sivadasan S et al. [12]. Perception and attitudes regarding the capability to report ADRs were significantly more positive in pharmacy students compared to medical students and this finding corresponds to other study conducted by Umair K et al. in Pakistan [13].

It is noteworthy to mention that only pharmacy students have more knowledge that SFDA is the governmental monitoring agency for ADRs in Saudi Arabia and the Saudi Vigilance System. This is an important concept that needs to be acknowledged by healthcare students in order to know how and where to report an ADR. The study findings showed that only one-third of pharmacy students knew that SFDA is the governmental monitoring agency for adverse drug reactions in Saudi Arabia which is lower than the rate reported by Alkayyal N et al. [18]. The present study revealed that about one-third of pharmacy students reported that the topic of PV is well covered in their curriculum, which is lower (55.6%) than the study done by Rajiah et al., among pharmacy students in Kuala Lumpur [19]. These findings from the present study show that the students have inadequate knowledge about Pharmacovigilance and it should be part of their college curriculum.

### Practice implications

Many practice implications emanate from the study findings. Given the significance of PV and ADR reporting in understanding and preventing drug-related problems, more training in the field of PV and ADRs is needed. It has been documented that lecture-based workshops regarding PV and ADRs reporting can improve the knowledge, attitude and practice of healthcare students [20]. Students should be familiarized with the ADR reporting and the ways for determining the causality and the severity of ADRs. Therefore, there is a need for integrating pharmacovigilance and adverse drug reaction reporting in the curriculum of healthcare schools. The World Health Organization (WHO) and the International Society of Pharmacovigilance (ISoP) (WHO-ISoP group) had generated core elements of a comprehensive PV curriculum to guide the PV integration into the healthcare schools’ curriculums [21]. These core elements should be integrated into the curriculum of healthcare students to improve their knowledge about PV.

### Strengths and limitations

This study provides a knowledge base on current knowledge, attitude, and perceptions towards pharmacovigilance and adverse drug reaction of healthcare students in Saudi Arabia. Moreover, a large sample size was collected to provide precise findings. Besides, this study investigated an important research question that can lead to improving the current pharmacovigilance education among healthcare students. Of course, any study is bound to have some limitations. First, the study participants consisted mostly of pharmacy and medicine students, meaning that our pool of participants was not much diverse academically speaking, which may reflect in the results. Second, the study is self-reported; therefore, we cannot eliminate recall bias. Also, we have not conducted a regression analysis, therefore, we did not control for confounding variables which is a major threat to the validity of inferences. Besides, the result of this study may not be generalizable to all healthcare school students in Saudi Arabia.

## Conclusions

The findings showed that pharmacy students have better knowledge and positive attitude and perception towards PV and ADRs reporting as compared to other healthcare students. Therefore, the study clearly describes the great need for integrating pharmacovigilance education in Saudi healthcare schools’ curriculums to prepare them for real-world practices and workplaces.

## Supplementary information

**Additional file 1.** Questionnaire

## Data Availability

The dataset supporting the conclusions of this article is available by request from the corresponding author.

## Abbreviations

PV: Pharmacovigilance
ADR: Adverse Drug Reaction
WHO: World Health Organization
