# Supplementary material for: Pharmacovigilance in healthcare education: students’ knowledge, attitude and perception: a cross-sectional study in Saudi Arabia
Source: BMC Med Educ. 2020 Jul 2;20:210. doi: 10.1186/s12909-020-02116-2 (PMC7331118; doi:10.1186/s12909-020-02116-2)
Supplement: Supplementary file 1 — Additional file 1. Questionnaire [file 12909_2020_2116_MOESM1_ESM.docx]

**Appendix I. Questionnaire**

***If you have read the research information and wish to participate, we would request that you complete the attached questionnaire and submit it. No name need to be written on the questionnaire as we wish to ensure anonymity of responses.***

**GENERAL INFORMATION**

**Q1. This section requests you to provide general information about yourself. This is important because different students may have different experiences and requirements for education about pharmacovigilance and Adverse drug reaction (ADR) reporting process, and we wish to understand individual training needs.**

**For questions below, please place a tick in the box next to the most suitable response.**

| 1. What is your Gender? | ☐ Male  ☐ Female |
| --- | --- |
| 1. In which healthcare school you are currently enrolled? | ☐ Medicine  ☐ Pharmacy  ☐ Nursing  ☐ Dentistry |
| 1. In which year of your study are you currently enrolled? (Including preparatory year\ common first year ) | ☐ 4^th^ ☐ 5^th^ ☐ 6^th^ ☐ Intern |
| 1. Is your college private or governmental? | ☐ Private  ☐ Government  **Please write the name of your University**………….. |
| 1. Have you ever had ADR or sensitivity to any medication? | ☐ Yes  ☐ No |
| 1. Do you have any family member who had ADR or sensitivity to any medication? | ☐ Yes  ☐ No |
| 1. Have you ever come across any patient during your training that had ADR or sensitivity to any medication? | ☐ Yes  ☐ No |
| 1. **If you answer 7 YES**, have you reported it? | ☐ Yes  ☐ No |

**PHARMACOVIGILANCE FACTS**

**Q2. Below are some questions about pharmacovigilance. Please choose ONE OR MORE answer/s for each question which you believe is/are correct.**

| 1. Pharmacovigilance is: ……………. | - **The science and activities relating to the detection, assessment, understanding and prevention of adverse effects** - The study related to safe, effective and economic use of medicines - Therapeutic Drug Monitoring |
| --- | --- |
| 1. The function of pharmacovigilance is\are:………. | - Improve patient care and safety in relation to the use of medicines - Identify the safety of the drugs - Detection of ADRs - **Identify the safety of the drugs, Detection of ADRs and Improve patient care and safety in relation to the use of medicines.** |
| 1. Adverse drug reaction (ADR) is …….. | - Any untoward medical occurrence that may present during treatment with a pharmaceutical product but which does not necessarily have a causal relationship with this treatment - **Any noxious change which is suspected to be due to a drug, occurs at dosed normally used in man, requires treatment or decrease in dose or indicates caution in future use of the same drug** - ADR is Adverse Event (AE) |
| 1. ADRs classified based on….. | - Onset of occurrence - Type of reaction - Severity - **Onset of occurrence, Type of reaction and severity** |
| 1. The scale used to assess the cause of ADRs named…... | - Probability scale - **Causality scale** - Severity scale |
| 1. Hypersensitivity reactions are ……to ADRs. | - **Related** - Unrelated |
| 1. The governmental monitoring agency for ADRs in Saudi Arabia is………….. | - Ministry of Health (MOH) - **Saudi Food and Drug Authority (SFDA)** - Saudi Arabia does not have a monitoring agency for ADRs |
| 1. In Saudi Arabia ……. | - No vigilance service available, so all ADRs should be submitted to WHO, using WHO online database - **Vigilance service is available through the National Pharmacovigilance and Drug Safety Center (Saudi Vigilance)** |
| 1. Which type of ADRs should be reported? | - **All ADRs should be reported regardless its severity** - Only serious adverse drug reaction should be reported |
| 1. ADRs that should be reported are related to………. | - Medications only - Medications + Over the counter products - Vaccines - Herbal products - Cosmetics - **Medications, Over the counter products, Vaccines, Herbal products, Cosmetics** |
| 1. Before reporting ADR, conformation that ADR is related to a particular drug is………. | - **Necessary** - **NOT** necessary |

**IMPORTANCE OF PHARMACOVIGILANCE AND ADR REPORTING**

**Q3. Following are some statements about YOUR thinking of pharmacovigilance importance and pharmacovigilance responsibility. Please indicate your opinion of a particular statement by ranking it along a scale of 1 (Strongly Agree) to 5 (Strongly Disagree). This can be done, by placing a tick in the column representing the number/rank you wish to select**

1. **Strongly agree,**
2. **Agree**
3. **Neutral\ Undecided**
4. **Disagree**
5. **Strongly disagree**

| **1= Strongly Agree 5= Strongly Disagree** | **(1)**  **Strongly agree** | **(2)**  **Agree** | **(3)**  **Neutral\ Undecided** | **(4)**  **Disagree** | **(5)**  **Strongly disagree** |
| --- | --- | --- | --- | --- | --- |
| 1. Reporting ADR make a significant contribution to reporting system |  |  |  |  |  |
| 1. Reporting ADR make a significant contribution to patient safety |  |  |  |  |  |
| 1. Reporting ADR is a responsibility of me |  |  |  |  |  |
| 1. Reporting ADR should be made compulsory for all health care professionals |  |  |  |  |  |

**PHARMACOVIGILANCE PRACTICE IN SAUDI MEDICAL COLLEGES**

**Q4. Please find below some statements about Pharmacovigilance and ADR reporting education in Saudi medical schools. Please choose Yes if your college has some practice that is similar to the one stated. If your college does not seem to have any of these practices in place, please choose No.**

| 1. Pharmacovigilance is well covered in your college curriculum | ☐ Yes  ☐ No |
| --- | --- |
| 1. Students in your college trained on how to report ADR | ☐ Yes  ☐ No |
| 1. Students in your college can perform ADR reporting during their clerkship | ☐ Yes  ☐ No |

**THE NEED FOR PHARMACOVIGILANCE AND ADR REPORTING EDUCATION**

**Q5. Education about Pharmacovigilance and ADR reporting implies some training/program/curriculum part that covered the basic knowledge and skills. We would like to ask you about your experience, your thoughts about being provided with such education, and opportunity that should be available to you.**

| 1. Have you received some form of pharmacovigilance education previously? | ☐ Yes  ☐ No  **If you answer No, go directly to 5** |
| --- | --- |
| 1. Do you feel you are adequately prepared to report ADR in your future practice? | ☐ Yes  ☐ No |
| 1. Do you believe that all medical students’ need education about pharmacovigilance and ADR reporting system? | ☐ Yes  ☐ No |
| 1. If student in your college be offered an opportunity to undertake education in pharmacovigilance and ADR reporting system, would you be willing to participate? | ☐ Yes  ☐ No |
